# Opposite Effects of Gene Deficiency and Pharmacological Inhibition of Soluble Epoxide Hydrolase on Cardiac Fibrosis

**DOI:** 10.1371/journal.pone.0094092

**Published:** 2014-04-09

**Authors:** Lijuan Li, Nan Li, Wei Pang, Xu Zhang, Bruce D. Hammock, Ding Ai, Yi Zhu

**Affiliations:** 1 Department of Physiology and Pathophysiology, Peking University Health Science Center, Beijing, China; 2 Department of Physiology, Tianjin Medical University, Tianjin, China; 3 Department of Entomology and Comprehensive Cancer Center, University of California Davis, Davis, California, United States of America; Northwestern University, United States of America

## Abstract

Arachidonic acid-derived epoxyeicosatrienoic acids (EETs) are important regulators of cardiac remodeling; manipulation of their levels is a potentially useful pharmacological strategy. EETs are hydrolyzed by soluble epoxide hydrolase (sEH) to form the corresponding diols, thus altering and reducing the activity of these oxylipins. To better understand the phenotypic impact of sEH disruption, we compared the effect of *EPHX2* gene knockout (*EPHX2* ^−/−^) and sEH inhibition in mouse models. Measurement of plasma oxylipin profiles confirmed that the ratio of EETs/DHETs was increased in *EPHX2* ^−/−^ and sEH-inhibited mice. However, plasma concentrations of 9, 11, 15, 19-HETE were elevated in *EPHX2* ^−/−^ but not sEH-inhibited mice. Next, we investigated the role of this difference in cardiac dysfunction induced by Angiotensin II (AngII). Both *EPHX2* gene deletion and inhibition protected against AngII-induced cardiac hypertrophy. Interestingly, cardiac dysfunction was attenuated by sEH inhibition rather than gene deletion. Histochemical staining revealed that compared with pharmacological inhibition, *EPHX2* deletion aggravated AngII-induced myocardial fibrosis; the mRNA levels of fibrotic-related genes were increased. Furthermore, cardiac inflammatory response was greater in *EPHX2* ^−/−^ than sEH-inhibited mice with AngII treatment, as evidenced by increased macrophage infiltration and expression of MCP-1 and IL-6. *In vitro*, AngII-upregulated MCP-1 and IL-6 expression was significantly attenuated by sEH inhibition but promoted by *EPHX2* deletion in cardiofibroblasts. Thus, compared with pharmacological inhibition of sEH, *EPHX2* deletion caused the shift in arachidonic acid metabolism, which may led to pathological cardiac remodeling, especially cardiac fibrosis.

## Introduction

Pathophysiological cardiac remodeling, characterized by cardiac hypertrophy and interstitial fibrosis, is one of the most common causes of heart failure [1], [2]. These pathophysiological changes of cardiac remodeling include hypertrophic growth and increased protein synthesis of cardiomyocytes [3] as well as hyperproliferation, collagen metabolism disorder and phenotype transforming of cardiac fibroblasts [4], which lead to contraction/dilation dysfunction and finally reduced compliance of the ventricle wall, all of which contribute to the development of heart failure. Adverse cardiac remodeling is always associated with inflammation, which plays a key role in the development and progression of cardiac fibrosis [5], [6]. Profibrotic stimuli such as Angiotensin II (AngII) or transforming growth factor β (TGF-β) treatment, hypertension and myocardial infarction lead to infiltration of inflammatory cells including macrophages, immune cells, neutrophils, mast cells and dendritic cells into the myocardium [7], [8], [9]. This infiltration releases numerous cytokines and chemokines, including interferon γ (IFN-γ), transforming growth factor α (TNF-α), TGF-β, and monocyte chemoattractant protein 1 (MCP-1), which may regulate further infiltration of inflammatory cells as well as cardiofibroblasts [10].

Arachidonic acid (ARA), derived from membrane phospholipids, can be metabolized by cyclooxygenases (COXs), lipoxygenases (LOXs), and cytochrome P450 enzymes (CYPs) to form biological active eicosanoids [11]. Several ARA metabolites are involved in the development of cardiac fibrosis associated with inflammation [10]. CYP enzymes metabolize ARA to multiple products including epoxyeicosatrienoic acids, consisting of 4 regioisomers (5,6-, 8,9-, 11,12-, 14,15-EET), or hydroxyl-eicosatetraenoic acids (HETEs), most notably 20-HETE, which are associated with inflammation [12], [13]. Eliminating or blocking 12/15- LOX reduced neutrophil recruitment and modulated neutrophil function response to endotoxin inhalation by decreasing 12-HETE and 15-HETE generation [14], [15], [16]. In addition, CYP4A- and CYP4F-derived 20-HETE is a proinflammatory mediator of endotoxin-induced acute systemic inflammation [17] involved in the development and/or progression of inflammatory cardiovascular diseases [18] by regulating monocyte/macrophage infiltration [19]. As compared with HETEs, EETs have vessel-dilation, myocardial-protective and anti-inflammatory effects [20], [21].

Soluble epoxide hydrolase (sEH) is the key enzyme hydrolyzing EETs to their corresponding dihydroxyeicosatrienoic acids (DHETs) and reducing the bioavailability of EETs [21]. Several generations of sEH inhibitors have been developed, and the administration of these drugs have beneficial effects on hypertension and cardiac dysfunction [22], [23]. Disruption of sEH gene (*EPHX2*) does not show alteration in basal blood pressure resulting from the shift in ARA metabolism to produce more 20-HETE in kidneys in both NIH and BI colonies [24], therefore sEH deletion and inhibition may have different effects. Our previous study demonstrated that sEH expression was induced by AngII in the rodent heart, and inhibition of sEH attenuated AngII-induced cardiac hypertrophy [25]. However, whether sEH is involved in AngII-induced cardiac fibrosis is still unknown. In this study, we compared the oxylipin profile with *EPHX2* deletion and sEH inhibition in mice to explore the effects of sEH in cardiac fibrosis and the underlying mechanisms. Our findings may help in understanding pathological cardiac remodeling and provide experimental evidence for sEH as a novel therapeutic target for cardiac fibrosis.

## Materials and Methods

### Ethics Statement and Animal Experiments

All animal experimental protocols were approved by the Peking University Institutional Animal Care and Use Committee. The investigation conformed to the Guide for the Care and Use of Laboratory Animals by the US National Institutes of Health (NIH Publication, 8th Edition, 2011). Mice with targeted disruption of *EPHX*2 gene (*EPHX2* ^−/−^) [26] were back-crossed onto a C57BL/6 genetic background for more than ten generations as previously described [24]. Male *EPHX2* ^−/−^ and their littermate control (*EPHX2^+/+^*) mice (8 weeks old, 20–25 g, Peking University Health Science Center Animal Department) were kept in a 12-hr light/dark cycle at a controlled room temperature and had free access to standard chow and tap water. On the day of surgery, *EPHX2* ^−/−^ and their littermate control mice were anaesthetized with a cocktail of ketamine (100 mg/kg intraperitoneal)/xylazine (5 mg/kg intraperitoneal) and implanted with a minipump (Alzet 1002) in the dorsal region to deliver AngII (1000 ng/kg/min for 14 days) or underwent a sham operation as a control. The adequacy of anesthesia was continually monitored by assessing reflexes and respiration. To examine the effect of sEH inhibition on AngII-induced hypertension, *EPHX2^+/+^* mice were divided into 4 groups for treatment(n≥6 mice per group): sham surgery+ vehicle group; AngII infusion(1000 ng/kg/min)+vehicle; AngII+TUPS (1- (1-methanesulfonyl-piperidin-4-yl)- 3- (4-trifluoromethoxy-phenyl) –urea); and TUPS only. TUPS was administrated by oral gavage daily at 4.0 mg/kg/day. After 3 days, the surgery was performed, and the mice were sacrificed on day 14th after the surgery. TUPS was prepared as previous described [25]. At the end of the experiment, mice received a cocktail of ketamine (100 mg/kg intraperitoneal)/xylazine (20 mg/kg intraperitoneal) for anesthesia and euthanized; hearts were removed, blotted, and weighed to determine the ratio of heart weight to body weight.

### Immunohistochemistry

Hearts were retrograde perfused with phosphate buffered saline (PBS) and fixed with 4% paraformaldehyde overnight, then embedded in paraffin, and serial left-ventricular (LV) sections 5 μm thick were cut along the longitudinal axis and stained with haematoxylin and eosin. Types I/III collagen in cardiac muscle was stained with picric acid–sirius red. For immunohistochemical staining of Mac3, for macrophages, after endogenous peroxidase was quenched and nonspecific reaction was blocked, sections were immunostained with a rabbit anti-Mac3 antibody (BD Pharmingen, USA) and horseradish peroxidase-conjugated secondary antibody (Life technology, USA). Diaminobenzidine tetrahydrochloride was used for color development. The resulting images were acquired by use of an Olympus CKX41 microscope and Olympus Micro software. Negative controls were species-matched IgG. The size of cardiomyocytes was determined from a mean of at least 200 cells by computer-assisted image analysis (NIH Image J). Measurements were taken by an observer blinded to the treatment groups. The extent of fibrosis was determined by use of an Axioplan 2 microscope (Zeiss) and MCID Elite 6.0 (Imaging Research), which analyzes data as a ratio of collagen area to total area.

### ELISA

Plasma interleukin 6 (IL-6) was measured by use of a mouse IL-6 ELISA kit (R&D Systems, Inc). Microtiter plates were read with use of a multiskan reader (Scientific Multiskan MK3, Thermo) at 450 nm (correction wavelength 540 nm).

### Neonatal Cardiofibroblasts in Culture

Murine neonatal cardiofibroblasts (NCFs) were isolated from 1- to 2-day old *EPHX2* ^−/−^ or their littermate control neonatal mice as described [27]. To isolate ventricles, neonates were euthanized by decapitation. We used a 60-min preplating procedure to obtain cardiofibroblasts and reduce the number of myocytes in cardiofibroblast culture. The purity of the obtained cardiofibroblast culture was confirmed to be more than 90% microscopically by characteristic cell morphologic features. NCFs were maintained in DMEM with 10% FBS, at 37°C in a 5% CO_2_ humidified incubator. NCFs were maintained in serum-free DMEM for 24 h before being incubated with AngII (1 μM) and/or TUPS (1 μM) for 24 h. We used NCFs from passage 2 for this study.

### Metabolomic Analysis

The blood of male *EPHX2* ^−/−^ and their littermate control (*EPHX2^+/+^*) mice was obtained when they were 10 weeks old. Plasma was extracted by solid-phase extraction (SPE). Before extraction, Waters Oasis-HLB cartridges were washed with methanol (1 mL) and MilliQ water (1 mL). Samples were spiked with internal standard mixture (5 ng for each internal standard). Plasma was loaded onto cartridges directly. Cartridges were washed with 1 mL of 5% methanol. The aqueous plug was pulled from the SPE cartridges by high vacuum, and SPE cartridges were further dried by low vacuum for about 20 min. Analytes were eluted into tubes with 1 mL methanol. The eluent was then evaporated to dryness. The residue was dissolved in 100 μl 30% acetonitrile. After vigorous mixing, samples were filtered into vials of an auto-sampler through a 0.22-μm membrane. Chromatographic separation involved an ACQUITY UPLC BEH C18 column (1.7 μm, 100×2.1 mm i.d.) consisting of ethylene-bridged hybrid particles (Waters, Milford, MA, USA). The column was maintained at 30°C and the injection volume was set to 10 μl. Solvent A was water and solvent B was acetonitrile. The gradient is given in Table S1. The mobile phase flow rate was 0.6 mL/min. Chromatography was optimized to separate ARA metabolites in 9 min. ARA metabolites were quantified by use of a 5500 QTRAP hybrid triple quadrupole linear ion trap mass spectrometer (AB Sciex, Foster City, CA, USA) equipped with a Turbo Ion Spray electrospray ionization (ESI) source. The mass spectrometer was operated using software Analyst 1.5.1. Analytes were detected using multiple reaction monitoring (MRM) scans in negative mode. The dwell time used for all MRM experiments was 25 ms. The ion source parameters were set as follows: CUR = 40 psi, GS1 = 30 psi, GS2 = 30 psi, IS = −4500 V, CAD = MEDIUM, TEMP = 500°C.

### Statistical Analysis

Data are presented as mean ± SEM. The significance of variability was evaluated by unpaired two-tailed Student’s *t* test or one-way ANOVA with a Bonferroni multiple comparison post-test (GraphPad software, San Diego, CA). Each experiment included triplicate measurements for each condition tested, unless indicated otherwise. P<0.05 was considered statistically significant.

### Others

Analysis of Cardiac Function by Echocardiography, Western Blot Analysis and Quantitative Real-Time RT–PCR (The sequences of primers are in Table S2). See Methods S1.

## Results

### sEH Deletion but not sEH Inhibition Shifted ARA Metabolism

**Figure 1:**
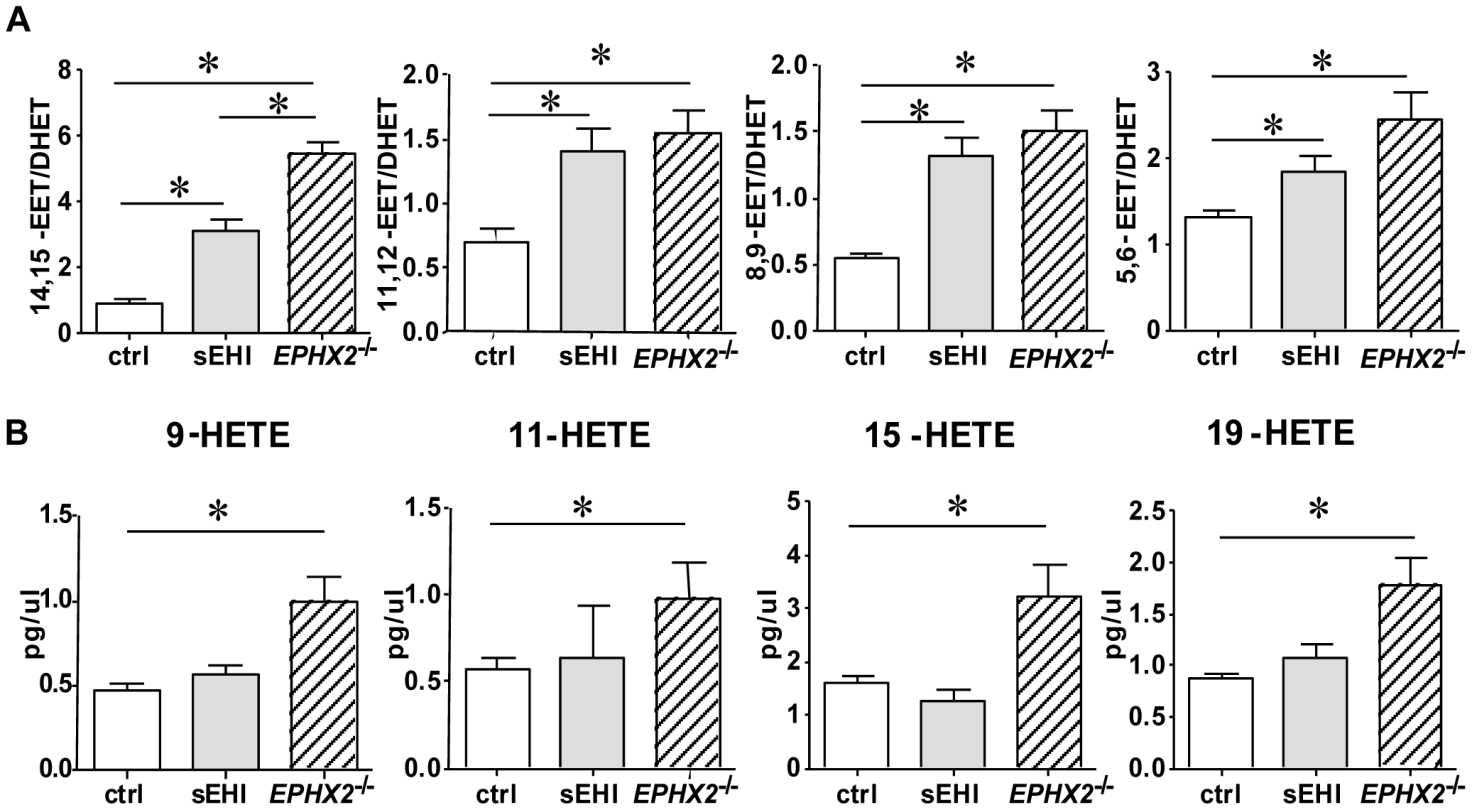
sEH deletion but not sEH inhibition upregulates the plasma level of several HETEs. Plasma concentration of ARA metabolites determined by LC-MS/MS. (A) Plasma ratio of EET to DHET. (B) Plasma concentration of 9-HETE, 11-HETE, 15-HETE and 19-HETE. Data are mean±SEM from at least 6 mice in each group (*, P<0.05).

To study the effect of *EPHX2* gene deletion and sEH inhibition on ARA metabolism, we first determined the plasma concentration of ARA metabolites by liquid chromatography-tandem mass spectrometry (LC-MS/MS). The plasma EET concentration was elevated to a similar extent in *EPHX2* ^−/−^ and sEH-inhibited mice, while that of DHETs, the metabolites of EETs, was lower in *EPHX2* ^−/−^ than sEH-inhibited mice (Table 1). As a result, the ratio of EET to DHET was greater in *EPHX2* ^−/−^ than sEH-inhibited mice. In particular, the ratio of 14, 15-EET:DHET was 1.9-fold higher in *EPHX2* ^−/−^ than sEH-inhibited mice (Figure 1A). Surprisingly, *EPHX2* gene deletion significantly increased 9-HETE (2.1-fold), 11-HETE (1.7-fold), 15-HETE (2.0-fold) and 19-HETE (2.0-fold) as compared with control but not sEH inhibition (Table 1 and Figure 1B). Therefore, although both *EPHX2* gene deletion and inhibition increased the ratio of EETs to DHETs, *EPHX2* gene deficiency rather than sEH pharmacological inhibition increased HETE production, which may result from the metabolic shift of ARA metabolism caused by excessive EET accumulation.

**Table 1:** Plasma arachidonic acid (ARA) metabolite concentration (pg/μl) determined by LC-MS/MS with soluble epoxide hydrolase (sEH) inhibition and deletion in mice.

Oxylipin	Vehicle	sEH inhibition	sEH deletion
Epoxygenase-dependent metabolism			
14,15-EET	0.412±0.015	1.120±0.039*	1.102±0.028*
11,12-EET	0.128±0.005	0.269±0.016*	0.257±0.010*
8,9-EET	0.151±0.006	0.384±0.018*	0.348±0.014*
5,6-EET	0.123±0.002	0.144±0.007	0.144±0.007
sEH-dependent metabolism			
14,15-DHET	0.515±0.012	0.346±0.015*	0.202±0.004* ^#^
11,12-DHET	0.204±0.005	0.196±0.009	0.162±0.002*
8,9-DHET	0.279±0.007	0.289±0.008	0.217±0.005*
5,6-DHET	0.096±0.002	0.070±0.002*	0.058±0.001*
CYP ω-hydrolase-dependent metabolism			
20-HETE	0.547±0.026	0.691±0.069	0.405±0.026
19-HETE	0.871±0.017	1.065±0.095	1.776±0.098*
18-HETE	0.549±0.013	0.570±0.028	0.537±0.016
17-HETE	0.230±0.006	0.235±0.006	0.222±0.007
16-HETE	0.155±0.004	0.236±0.014*	0.153±0.003
CYP allylic-oxidase-dependent metabolism			
11-HETE	0.564±0.015	0.642±0.103	0.980±0.053*
9-HETE	0.476±0.01	0.567±0.017	0.992±0.056*
LOX-dependent metabolism			
15-HETE	1.590±0.034	1.283±0.076	3.2±0.175*
12-HETE	15.292±0.944	16.565±1.564	13.549±0.666
8-HETE	0.541±0.025	0.57±0.049	0.663±0.038
5-HETE	1.379±0.053	1.72±0.16	1.507±0.056
15-oxo-ETE	NP	NP	NP
5-oxo-ETE	NP	NP	NP
LTB4	NP	NP	NP
LXA4	NP	NP	NP
COX-dependent metabolism			
TXB2	0.038±0.001	0.051±0.003	0.041±0.002
PGE2	0.057±0.007	0.044±0.007	0.046±0.004
PGD2	NP	NP	NP
PGB2	NP	NP	NP
PGF2a	NP	NP	NP
PGJ2	0.131±0.008	0.108±0.001	0.15±0.011
15-deoxy-PGJ2	NP	NP	NP
6-keto-PGF1a	0.421±0.019	0.551±0.036	0.364±0.011

*p<0.05 compared with vehicle; ^#^p<0.05 compared with with sEHI.

Data are mean±SEM from at least 6 mice in each group. NP: No peak; CYP: cytochrome P450 enzymes; COX: cyclooxygenase, LOX: lipoxygenase, PG: prostaglandin.

### Both sEH Deletion and Inhibition Protected Against Angll-induced Cardiac Hypertrophy

Because the level of 4 HETEs increased in *EPHX2* ^−/−^ mice was found associated with vascular remolding by a pro-inflammatory effect [15], [28], [29], [30], [31], we next explored the different physiological effects of *EPHX2* gene deletion and inhibition in a mouse cardiac hypertrophy model. *EPHX2* ^−/−^ and wild-type mice received sustained infusion of AngII (1000 ng/kg/min) via an implanted minipump for 14 days. SBP was measured every other day by tail cuff plethysmography and SBP was significantly increased from 100 mmHg to 150 mmHg with AngII infusion in wild-type mice, which was attenuated by *EPHX2* deficiency and treatment with the sEH inhibitor TUPS (oral gavage, 4.0 mg/kg/day) to about 120 mmHg (data not shown). Moreover, both *EPHX2* deficiency and sEH inhibition protected against AngII-induced cardiac hypertrophy, which was assessed by ratio of heart weight to body weight, left ventricular wall thickness (Table 2 and Table 3), relative cell area of cardiomyocytes and expression of the hypertrophy biomarker atrial natriuretic peptide (ANP) and β-isoform of myosin heavy chain (β-MHC) (Figure S1).

**Table 2:** sEH inhibition blocks Angiotensin II (AngII)-induced cardiac hypertrophy and change in cardiac function in mice.

	sham		AngII	
	vehicle	sEHI	vehicle	sEHI
HW/BW (mg/g)	5.10±0.09	5.09±0.13	5.80±0.13*	5.26±0.10^#^
LVW/BW (mg/g)	2.65±0.19	3.08±0.08	3.52±0.15*	2.98±0.18^#^
LVPW;d (mm)	0.69±0.02	0.75±0.02	0.88±0.04*	0.83±0.06
LVPW;s (mm)	0.99±0.04	1.04±0.04	1.31±0.05*	1.12±0.06^#^
LVAW;d (mm)	0.64±0.02	0.71±0.02	0.95±0.05*	0.75±0.02^#^
LVAW;s (mm)	0.88±0.04	1.01±0.03	1.31±0.07*	1.08±0.03^#^
LVEDV (μl)	55.29±2.14	54.95±5.78	37.47±2.74*	50.30±5.44^#^
LVESV (μl)	25.02±2.08	19.91±1.60	11.65±1.47*	18.60±2.67^#^
LVFS (%)	29.37±2.12	33.35±1.80	40.68±1.37*	34.57±2.66^#^
LVEF (%)	56.35±2.86	62.51±2.35	73.23±2.70*	62.13±2.98^#^

Data are mean±SEM; * p<0.05 compared with vehicle sham;^ #^p<0.05 compared with vehicle AngII.

**Table 3:** sEH deletion protects against AngII-induced cardiac hypertropy but does not affect the change in cardiac function.

	sham		AngII	
	sEH^+/+^	sEH^−/−^	sEH^+/+^	sEH^−/−^
HW/BW (mg/g)	4.91±0.11	5.23±0.17	6.14±0.23*	5.43±0.11^#^
LVW/BW (mg/g)	1.93±0.08	2.27±0.24	2.99±0.18*	2.42±0.15^#^
LVPW;d (mm)	0.57±0.02	0.61±0.02	0.88±0.07*	0.64±0.05^#^
LVPW;s (mm)	0.78±0.03	0.86±0.02	1.16±0.06*	0.88±0.07^#^
LVAW;d (mm)	0.55±0.03	0.63±0.02*	0.95±0.08*	0.71±0.08^#^
LVAW;s (mm)	0.78±0.04	0.85±0.01	1.30±0.12*	0.92±0.09^#^
LVEDV (μl)	53.19±2.93	57.25±6.27	41.07±3.10*	47.17±5.03
LVESV (μl)	26.34±1.57	25.37±3.85	15.71±1.86*	21.99±4.07
LVFS (%)	26.14±1.17	29.06±1.45	32.90±2.05*	28.69±2.52
LVEF (%)	51.24±1.80	56.48±2.29	62.29±2.79*	55.80±3.40

Data are mean±SEM. * p<0.05 compared with sEH^+/+^ sham; ^#^p<0.05 compared with sEH^+/+^ AngII.

BW: body weight; HW: heart weight; LVW: left ventricular weight; LVPWs: LV posterior wall thickness at systole; LVPWd: LVPW at diastole; LVAWd: LV anterior wall thickness at diastolic; LVAWs: LVAW at systole; LVESV: LV end-systolic volume; LVFS: LV fractional shortening; LVEF: LV ejection fraction.

### Deletion of *EPHX2* Aggravated AngII-induced Cardiac Fibrosis

**Figure 2:**
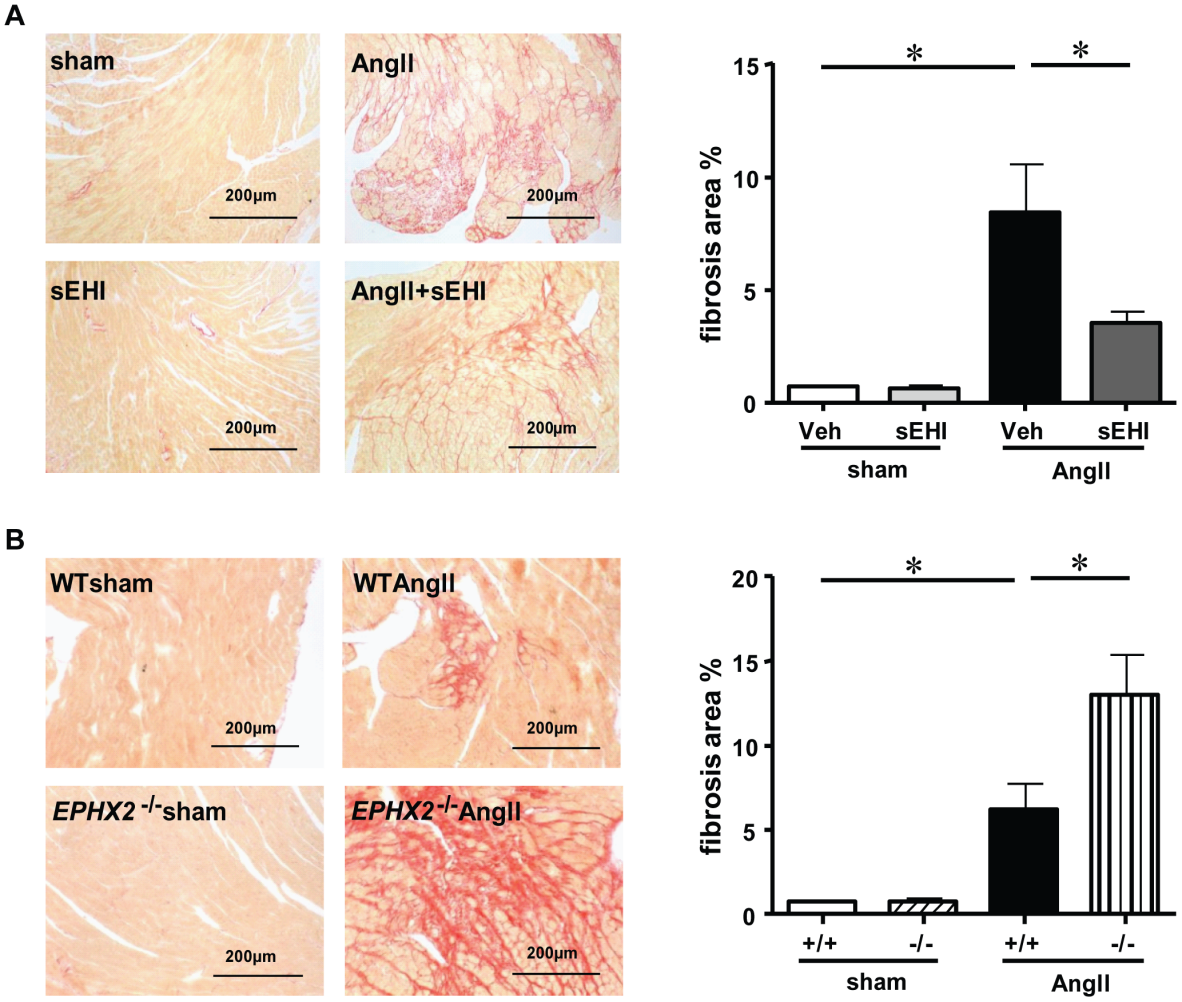
sEH deletion and inhibition have opposite effects on AngII-induced cardiac fibrosis. (A, B) Cross-sections of mouse left ventricles were counterstained with picric acid–sirius red for fibrosis and quantification. Data are mean±SEM from at least 6 mice in each group (*, *P*<0.05). Sham: sham infusion; sEHI: sEH inhibition; −/−: *EPHX2* gene deletion.

Although sEH deletion and inhibition have similar effects on AngII-induced hypertension and cardiac hypertrophy, their effects on cardiac function were opposite. AngII infusion decreased left-ventricular (LV) end-diastolic volume and LV end-systolic volume and increased LV fractional shortening and LV ejection fraction (Table 2 and Table 3). These data suggest that the heart function was in a compensation period after 14 days of AngII infusion. When we examined the involvement of sEH in cardiac function, sEH inhibition attenuated the effects of AngII (Table 2). Interestingly, as compared with sEH inhibition, *EPHX2* deletion could not reverse the AngII-induced cardiac dysfunction (Table 3). To further analyze the phenotype of the cardiac dysfunction, we measured cardiac fibrosis in those two models by picric acid–sirius red staining. Compared with vehicle treatment, cardiac collagen deposition was prevented by 42% by administration of TUPS (Figure 2A). However, AngII-induced myocardial fibrosis was aggravated in *EPHX2* ^−/−^ mice (Figure 2B).

### sEH Deletion and Inhibition had Opposite Effects on the Expression of Genes Related to Collagen Synthesis in the Heart

**Figure 3:**
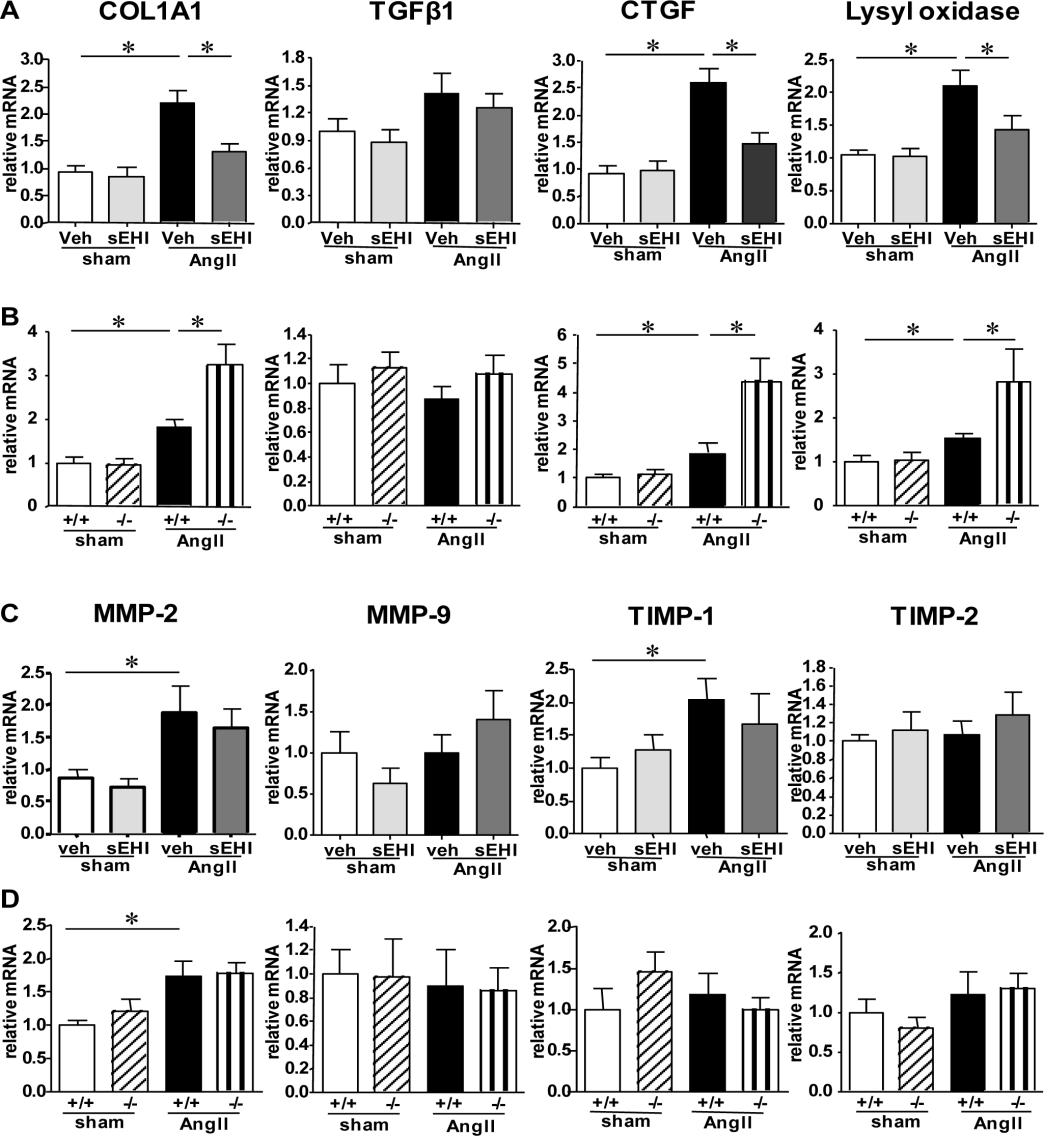
Expression of cardiac fibrosis-related genes in sEH deleted and inhibited mice with AngII infusion. (A, B) Real-time PCR analysis of mRNA expression of collagen-synthesis–related genes. COL1A1: collagen type 1, alpha 1; TGF-β1: transforming growth factor β1; CTGF: connective tissue growth factor. (C, D) Real-time PCR analysis of mRNA expression of collagen-degradation–related genes. MMP2/9, matrix metalloproteinase 2/9; TIMP1/2, tissue inhibitors of metalloproteinase-1/2. Data are mean±SEM relative to that of GAPDH from at least 6 mice in each group (*, P<0.05).

We investigated the impact of *EPHX2* gene deletion and inhibition on the expression of fibrosis-related genes. AngII infusion increased the mRNA level of both collagen synthesis genes such as collagen I, pro-fibrotic cytokine connective tissue growth factor (CTGF), and Lysyl oxidase (Figure 3A, B), as well as collagen degradation genes such as matrix metalloproteinase 2 (MMP2) and tissue inhibitor of metalloproteinase 1 (TIMP-1) (Figure 3C, D). The mRNA levels of collagen I, CTGF, and Lysyl oxidase were reduced to 60%, 56%, and 68%, respectively, by sEH inhibition as compared with AngII infusion alone (Figure 3A). In contrast, *EPHX2* ^−/−^ mice showed significantly increased level of these genes, by 78%, 134%, and 83%, respectively (Figure 3B). Neither *EPHX2* deletion nor sEH inhibition affected the expression of collagen-degradation–related genes, including MMP-2/9 and their tissue inhibitors (TIMP-1/2) (Figure 3C, D), which suggests that the opposite effect of sEH deletion and inhibition on AngII-induced cardiac fibrosis is via influencing collagen synthesis rather than degradation.

### *EPHX2* Gene Deletion Aggravates AngII-induced Cardiac Inflammation

**Figure 4:**
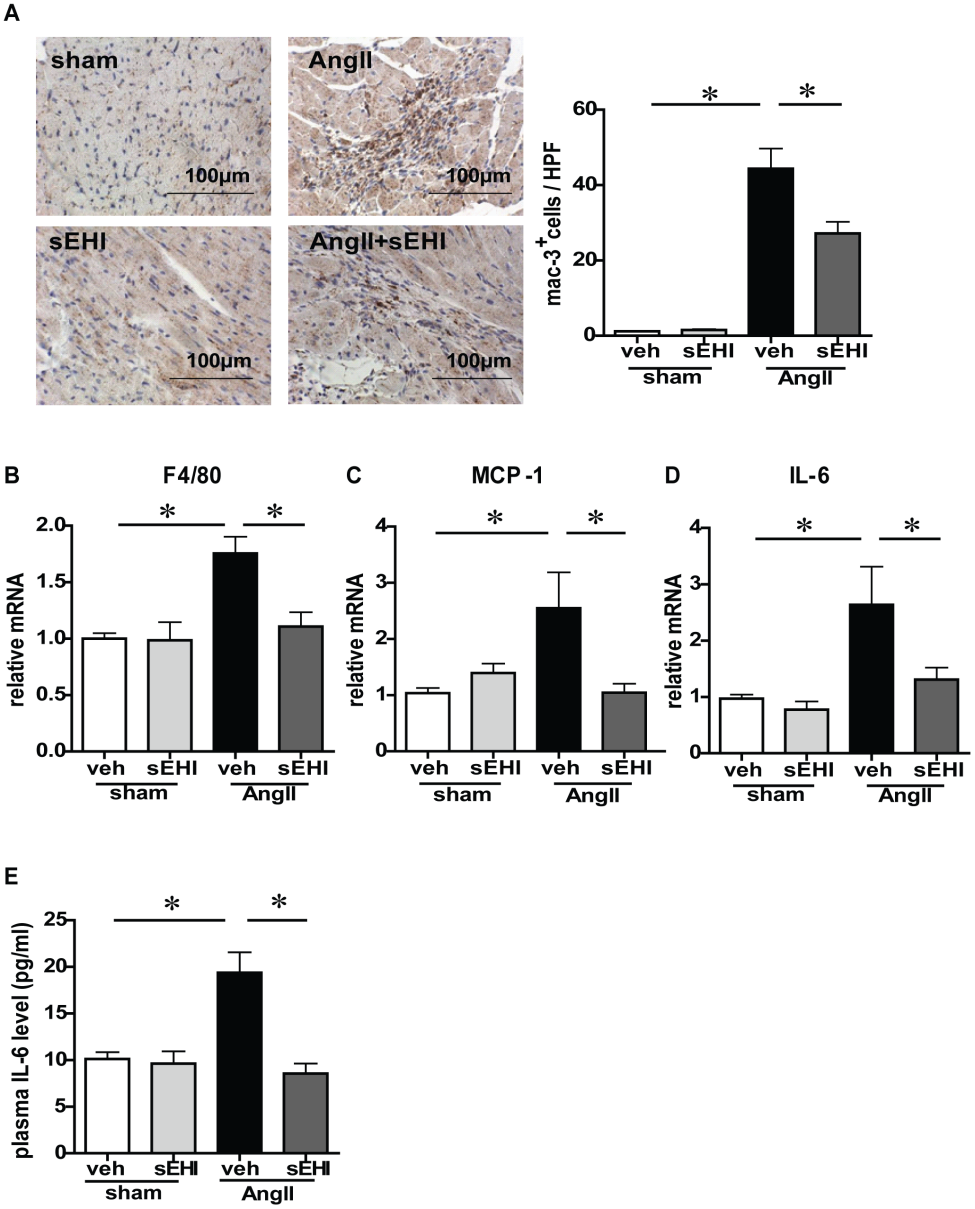
sEH inhibition blocks AngII-induced upregulation of chemokines and cytokines. (A) Cross-sections of mouse left ventricle underwent immunohistochemistry staining with anti-mac3 antibody and quantification. The mRNA level of (B) F4/80, (C) monocyte chemoattractant protein 1 (MCP-1), and (D) interleukin 6 (IL-6) in LV tissue. (E) ELISA of plasma IL-6 level. Data are mean±SEM from at least 6 mice in each group (*, *P*<0.05).

sEH was reported by Spector et al to be the major enzyme involved in the degradation of EETs which played an important role in myocardial inflammation [20], [21]. To test whether sEH affects AngII-induced collagen synthesis process by influencing cardiac inflammation, we measured inflammation in the myocardium *in vivo*. The infiltration of macrophages was determined by immunohistochemistry staining with anti-Mac3 antibody. As compared with control mice, AngII infusion caused an increased number of Mac3^+^ cells infiltrating into heart tissue, and the phenotype was reduced by TUPS treatment (Figures 4A). Consistently, TUPS significantly decreased the mRNA level of F4/80 to 67% (Figure 4B) and the expression of inflammatory factors such as MCP-1 to 41% (Figure 4C) and IL-6 to 50% (Figure 4D) in LV tissue as compared with vehicle-treated AngII-infused mice. Moreover, elevated plasma level of IL-6 with AngII infusion was suppressed by TUPS treatment (Figure 4E). Therefore, sEH inhibition attenuated cardiac inflammation induced by AngII.

**Figure 5:**
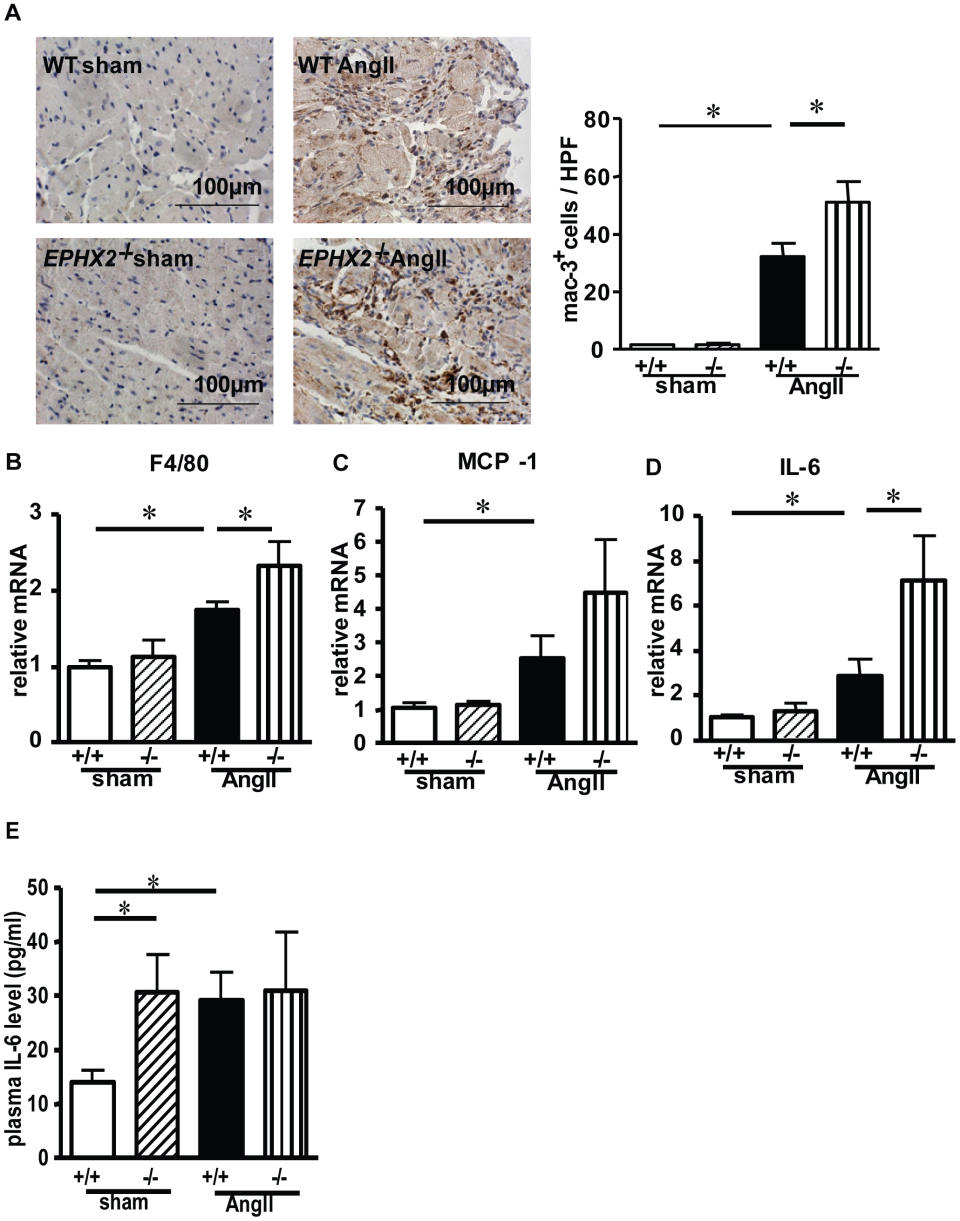
*EPHX2* gene deletion aggravates AngII-induced cardiac inflammation. (A) Cross-sections of mouse left ventricle underwent immunohistochemistry staining with anti-mac3 antibody and quantification. The mRNA level of (B) F4/80, (C) MCP-1, and (D) IL-6 in LV tissue. (E) ELISA of plasma IL-6 level. Data are mean±SEM from at least 6 mice in each group (*, *P*<0.05).

We next evaluated the function of *EPHX2* gene deletion in cardiac inflammation. Surprisingly, AngII-induced macrophage accumulation in LV tissue was aggravated in *EPHX2* ^−/−^ mice. Mac3^+^ cells in the hearts of AngII-infused *EPHX2* ^−/−^ mice was 158% that of control mice (Figures 5A), and the mRNA levels of F4/80 (Figure 5B), MCP-1 (Figure 5C) and IL-6 (Figure 5D) in LV tissue of AngII-infused *EPHX2* ^−/−^ mice were further increased by 34%, 76%, and 153%, respectively. Different from the mRNA level, basal level of plasma IL-6 in *EPHX2* ^−/−^ mice was 2.2 folds of WT control mice (Figure 5E), and *EPHX2* deficiency did not further increased the levels of plasma IL-6 in AngII-infused mice which suggested that local IL-6 level rather than circulation level determined cardiac inflammation. We also tested other inflammatory cytokines such as IFNγ, TNFα and IL-1β, but there was no significant change in our model (Figure S2). Thus, sEH deletion and inhibition had opposite effects on cardiac inflammation and macrophage accumulation, which may contribute to the formation of cardiac fibrosis.

### Effect of sEH on AngII-induced Production of Inflammatory Factors in Cardiofibroblasts *in vitro*

**Figure 6:**
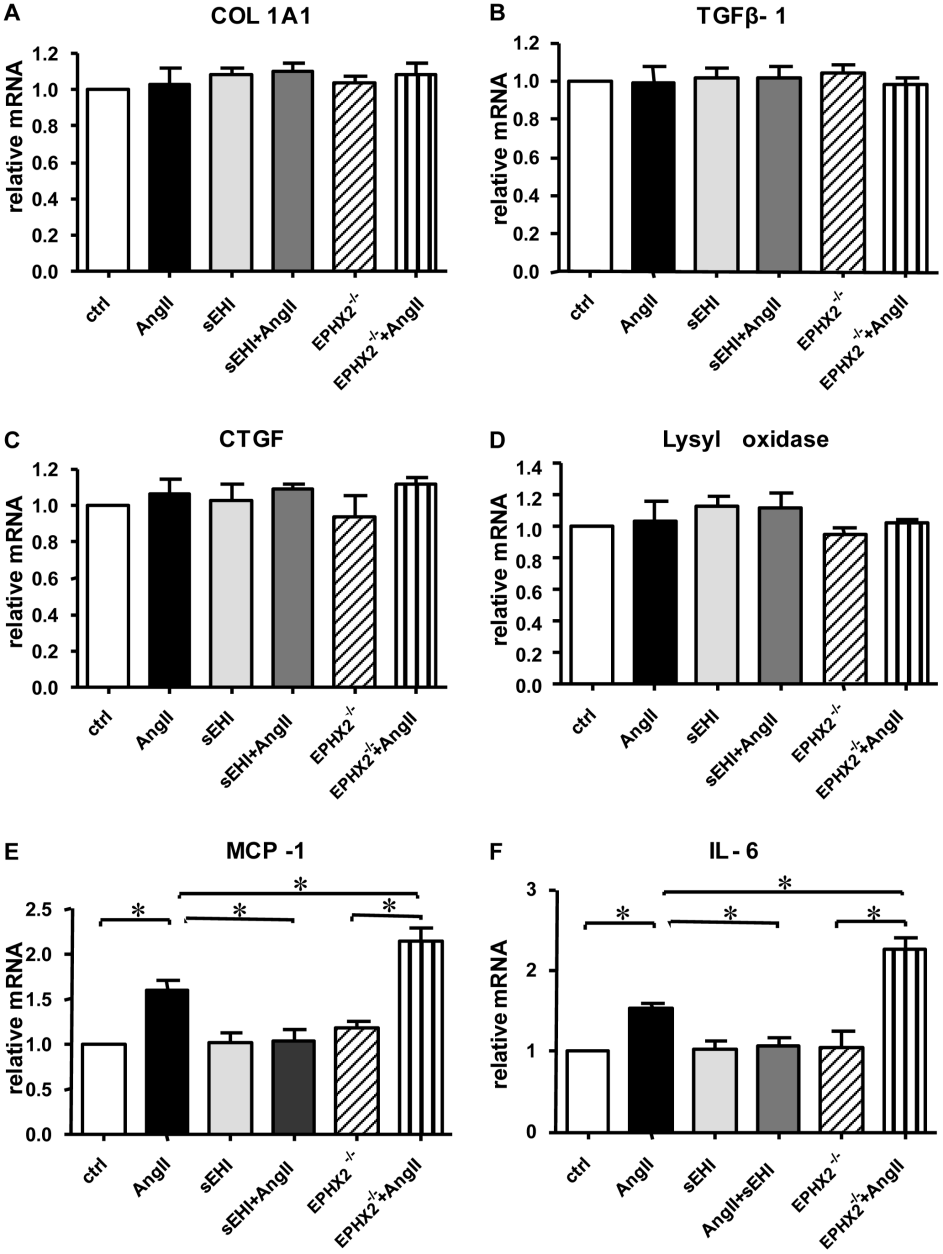
The effect of sEH on collagen synthesis and inflammatory factors expression in cardiofibroblasts *in vitro*. Neonatal cardiofibroblasts from *EPHX2^−/−^* or control mice were treated with 1 μM AngII and/or 1 μM sEH inhibitor (TUPS) for 24 h. Real-time PCR analysis of mRNA expression of collagen-synthesis–related genes and inflammatory factors. (A) COL1A1; (B) TGFβ1; (C) CTGF; (D) lysyl oxidase; (E) MCP-1 and (F) IL-6. Data are mean±SEM from 3 independent experiments (*, *P*<0.05).

We tested the mechanism of the difference between sEH deficiency and inhibition in an *in vitro* setting. As a latest generation sEH inhibitor, sEH activity was reduced dramatically by TUPS in cultured cardiac cells [25], [32]. We isolated cardiofibroblasts from wild-type or *EPHX2* ^−/−^ mice and treated the cells with AngII and/or TUPS for 24 hr, then measured the expression of collagen-synthesis–related genes and inflammatory factors. Unexpectedly, the collagen synthesis function of cardiofibroblasts was not influenced by AngII or sEH deletion/inhibition (Figure 6A–D). However, the change in levels of inflammatory factors was consistent with *in vivo* data. Administration of AngII for 24 hr significantly increased the mRNA level of MCP-1 to 161% (Figure 6E) and IL-6 to 152% (Figure 6F) which was attenuated to control level by sEH inhibition. In contrast, *EPHX2* deficiency further elevated the mRNA levels of MCP-1 and IL-6 to 214% and 227%, respectively (Figure 6E, F). Therefore, sEH participated in the process of cardiac fibrosis systemically, including via production of cardiofibroblast inflammatory factors and macrophage infiltration.

## Discussion

Arachidonic acid (ARA) is a free fatty acid derived from membrane phospholipids by phospholipase A_2_ (PLA_2_) and other enzymes. It can be metabolized by COXs, LOXs, and CYPs to form many biological active eicosanoids [11]. Some ARA metabolites such as EETs and PGI_2_ have anti-inflammatory and cardioprotective roles [33], [34], but many are pro-inflammatory and pro-fibrotic eicosanoids [35], [36], [37], [38]. In this study, we investigated the ARA metabolism in the blood of sEH inhibited and deficiency mice. Our LC-MS/MS data showed a higher ratio of EETs to DHETs with sEH deletion than inhibition and increased plasma concentration of 9-HETE, 11-HETE, 15-HETE and 19-HETE, which potentially eliminated the beneficial effect of EETs. Although we did not detect the change of 20-HETE, we cannot exclude it because of the possible limitation of our methods.

sEH is a homodimer consisting of two domains with two distinct activities: the N-terminal domain phosphatase activity and C-terminal epoxide hydrolase activity [39]. The C-terminal is the site of the epoxy-fatty acid hydrolysis which the sEH inhibitors including TUPS are against. Although the role of phosphatase domain has yet to be fully uncovered, N-terminal may play a role in regulating cholesterol synthesis in liver [40], [41] and altering the phosphorylation of endothelial nitric oxide synthase (eNOS) in endothelial cells [42]. In our study, different from partial inhibition of sEH by sEH inhibitor, global *EPHX2* deficiency resulted in a total defect in the sEH metabolic pathway with higher ratio of EETs to DHETs, and the high EETs levels caused an adaption by shifting ARA metabolism to other proinflammatory pathways. Consistent with our study, Luria et al indicated that *EPHX2*-null mice maintained normal basal blood pressure and reduced hypotensive effects of LPS challenge by increasing renal 20-HETE production through a feedback effect on CYP4A [24]. Since beneficial effects of sEH inhibitor are dependent on C-terminal, the loss of N-terminal in *EPHX2* ^−/−^ mice may contribute to the opposite phenomenon observed in sEH deficient and inhibited mice. In *EPHX2* ^−/−^ mice, lysophosphatidic acids (LPA) hydrolysis activity is 99% less than wild type mice [43], suggesting LPAs are the best nature substrates for sEH N-terminal. By binding to LPA receptors, LPA induced COX-2 expression and modulates proinflammatory gene expression [44]. As an inflammation mediator, recent study implicated crossover of the 5-LOX and COX-2 pathways as an alternative biosynthetic route of diHETEs from HETEs [45], which may explain the shift of AA metabolic profile in *EPHX2* ^−/−^ mice. In addition, as Luria et al shown, CYP enzymes may also be directly modulated by EETs overload which allow organisms to reduce the excess EETs and maintain homeostatic control of critical phenotypic characteristics [24]. Different from pharmacological inhibition of sEH by TUPS, although *EPHX2* deletion resisted the AngII induced hypertension and cardiac hypertrophy, it aggravated the cardiac fibrosis, which has been proposed as a major determinant leading to both cardiac systolic and diastolic dysfunction [46], [47] and contribute to the deterioration of cardiac dysfunction.

Our previous studies showed that in AngII-infused rat model, the sEH inhibitor TUPS could repress hypertension and the hypertrophic process [25]. However, the involvement of sEH in pathological cardiac remodeling induced by AngII, especially in the interstitial fibrosis process, was still unclear. In the current study, we evaluated cardiac fibrosis in sEH deletion and inhibited mice. Consistent with the study by Sirish and colleagues, sEH inhibition prevented AngII-induced interstitial fibrosis [48]. Surprisingly, we observed increased cardiac fibrosis in *EPHX2* deletion mice. As compared with reduced collagen-synthesis gene expression caused by sEH inhibition, *EPHX2* deficiency further upregulated collagen I and pro-fibrotic factors induced by AngII. However, our *in vitro* experiments showed that sEH did not directly affect the expression of fibrosis genes in myofibroblasts. We found that opposite to *EPHX2* deficiency, administration of the sEH inhibitor TUPS effectively attenuated MCP-1 and IL-6 expression which may result in decreased macrophage accumulation. Many studies showed that inflammation plays a key role in the development and progression of cardiac fibrosis [5], [6]. The inflammatory factors secreted by cardiofibroblasts activate inflammatory cells such as macrophages, lymphocytes, and mast cells. Inflammatory cells infiltrating into the myocardium release numerous inflammatory factors, including IFNγ, TNFα, TGFβ and MCP-1, which further recruit inflammatory cells as well as cardiofibroblasts [10]. The association of cardiac fibrosis and inflammatory response suggested that opposite effect of sEH deletion and inhibition on AngII-induced cardiac fibrosis is inflammation-dependent which may be caused by different ARA metabolism as we stated before.

In conclusion, we provide novel insights into the role of sEH in regulating AngII-induced MCP-1 and IL-6 expression and cardiac fibrosis. Different from the beneficial effect of partial sEH disruption by pharmacological inhibition, the compensation effect of total *EPHX2* deficiency shifted ARA metabolism to ω-hydrolase–LOX pathways, increased the level of pro-inflammatory factor HETEs and eliminated the anti-inflammation and cardioprotective effect of EETs. Increased MCP-1 and IL-6 expression in *EPHX2* deficiency mice may promote AngII-induced macrophage infiltration which increased ECM synthesis and secretion in cardiofibroblasts. Our results suggest that sEH is involved in pathological cardiac remodeling, especially cardiac fibrosis, depending on the way of sEH disruption. These findings may reveal a novel effect of sEH in cardiac fibrosis and have clinical significance for treatment of cardiac remodeling.

## Supporting Information

Figure S1 **Both sEH deletion and inhibition protected against AngII-induced cardiac hypertrophy.** (A, D) Cross sections of mouse left ventricles were stained with hematoxylin and quantification of the relative cell area of cardiomyocytes was performed. (B, E) Representative images of echocardiography. (C, F) Real-time PCR analysis of the mRNA level of atrial natriuretic protein (ANP) and β-myosin heavy chain (β-MHC) in left-ventricular (LV) tissue. Data are mean±SEM from at least 6 mice in each group (*P<0.05)**.** Sham, sham infusion; sEHI, sEH inhibition; −/−, *EPHX2* gene deficiency.
(TIF)

Figure S2 **Neither sEH inhibition nor** ***EPHX2*** **null affected the expression of several inflammation cytokines.** Real-time PCR analysis of the mRNA level of interferon γ (IFNγ), tumor necrosis factor α (TNFα) and interleukin-1β (IL-1β) in LV tissue. Data are mean ± SEM relative to that of GAPDH from at least 6 mice in each group (*, P<0.05).
(TIF)

Table S1 **LC gradient.**
(DOC)

Table S2 **Primers used for real-time PCR.**
(DOC)

Methods S1 (DOC)
