# Supplementary figures and images for: Opposite Effects of Gene Deficiency and Pharmacological Inhibition of Soluble Epoxide Hydrolase on Cardiac Fibrosis
Source: PLoS One. 2014 Apr 9;9(4):e94092. doi: 10.1371/journal.pone.0094092 (PMC3981766; doi:10.1371/journal.pone.0094092)

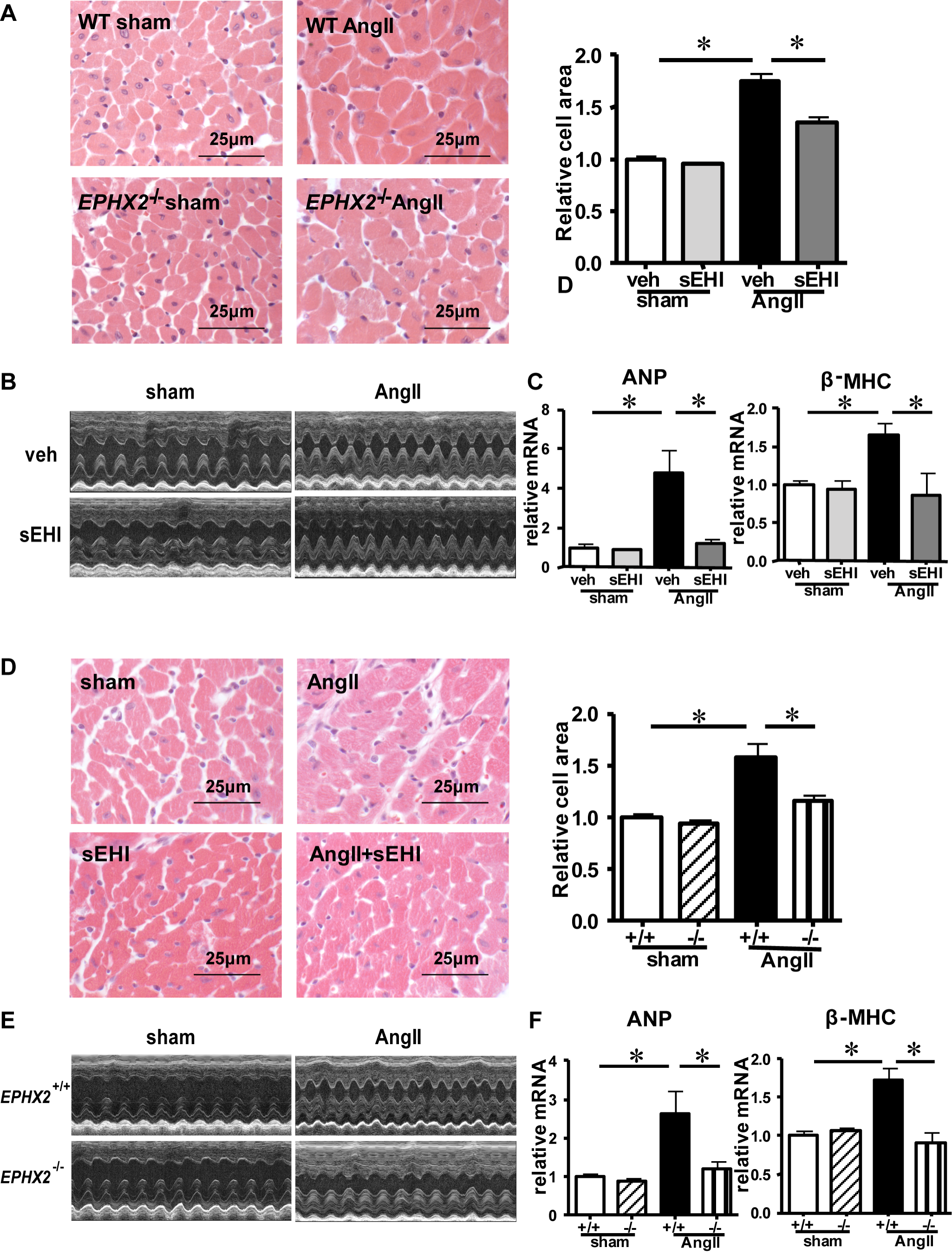

Supplement: Figure S1 — Both sEH deletion and inhibition protected against AngII-induced cardiac hypertrophy. (A, D) Cross sections of mouse left ventricles were stained with hematoxylin and quantification of the relative cell area of cardiomyocytes was performed. (B, E) Representative images of echocardiography. (C, F) Real-time PCR analysis of the mRNA level of atrial natriuretic protein (ANP) and β-myosin heavy chain (β-MHC) in left-ventricular (LV) tissue. Data are mean±SEM from at least 6 mice in each group (*P<0.05). Sham, sham infusion; sEHI, sEH inhibition; −/−, EPHX2 gene deficiency. (TIF) [file pone.0094092.s001.tif]

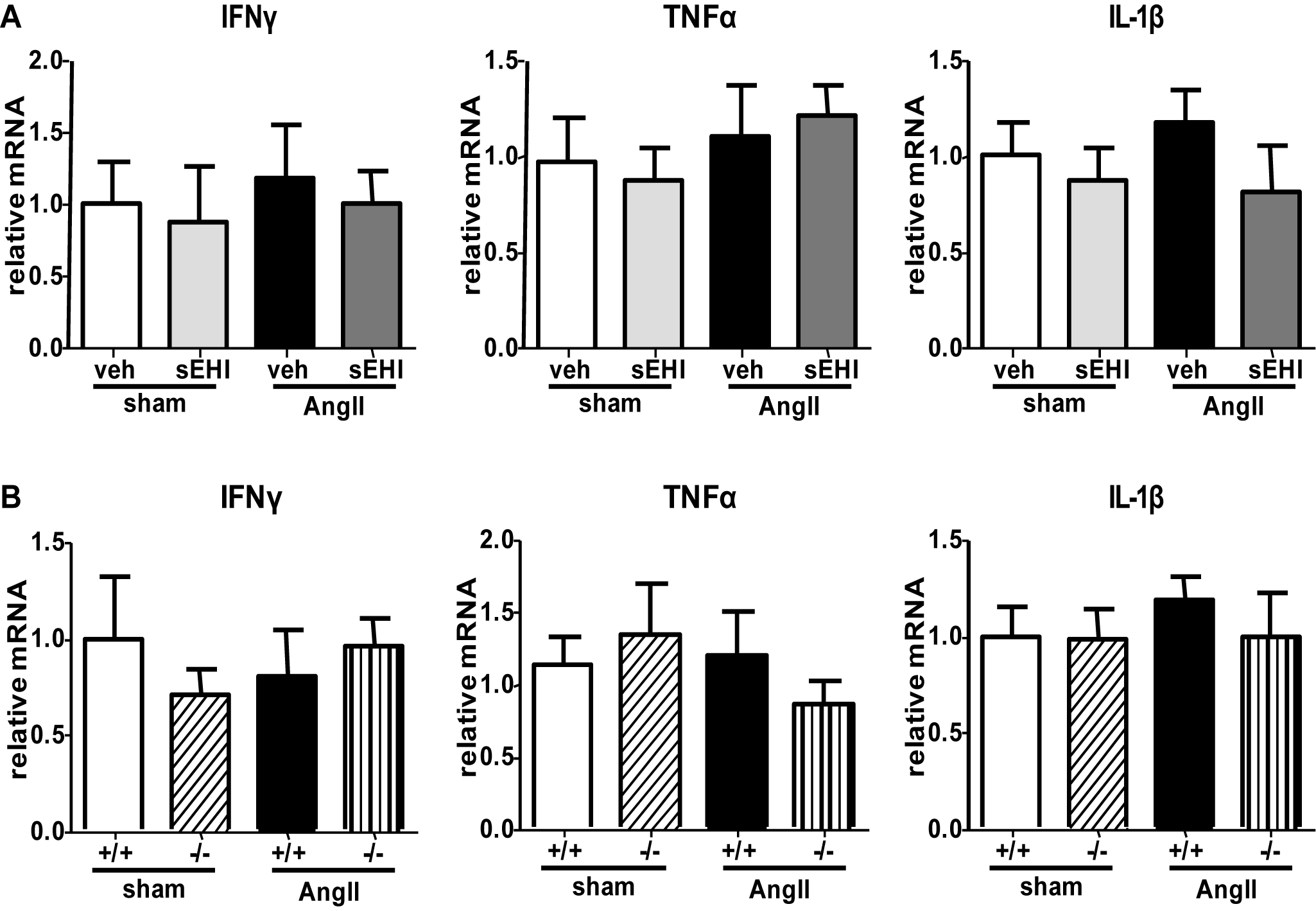

Supplement: Figure S2 — Neither sEH inhibition nor EPHX2 null affected the expression of several inflammation cytokines. Real-time PCR analysis of the mRNA level of interferon γ (IFNγ), tumor necrosis factor α (TNFα) and interleukin-1β (IL-1β) in LV tissue. Data are mean ± SEM relative to that of GAPDH from at least 6 mice in each group (*, P<0.05). (TIF) [file pone.0094092.s002.tif]
