# Supplementary material for: Opposite Effects of Gene Deficiency and Pharmacological Inhibition of Soluble Epoxide Hydrolase on Cardiac Fibrosis
Source: PLoS One. 2014 Apr 9;9(4):e94092. doi: 10.1371/journal.pone.0094092 (PMC3981766; doi:10.1371/journal.pone.0094092)
Supplement: Table S1 — LC gradient. (DOC) [file pone.0094092.s003.doc]

**Table S1. LC gradient**

| **Time (min)** | **A %** | **B %** |
| --- | --- | --- |
| 0 | 70 | 30 |
| 1.5 | 60 | 40 |
| 6.5 | 40 | 60 |
| 7.6 | 20 | 80 |
| 8.6 | 20 | 80 |
| 8.8 | 70 | 30 |
| 9.0 | 70 | 30 |

Solvent A was water and solvent B was acetonitrile.
