# Supplementary material for: Opposite Effects of Gene Deficiency and Pharmacological Inhibition of Soluble Epoxide Hydrolase on Cardiac Fibrosis
Source: PLoS One. 2014 Apr 9;9(4):e94092. doi: 10.1371/journal.pone.0094092 (PMC3981766; doi:10.1371/journal.pone.0094092)
Supplement: Table S2 — Primers used for real-time PCR. (DOC) [file pone.0094092.s004.doc]

**Table S2**. Primers used for real-time PCR

| **Gene** | **Forward primer** | **Reverse primer** |
| --- | --- | --- |
| GAPDH | 5’-AATGCATCCTGCACCACC-3’ | 5’-ATGCCAGTGAGCTTCCCG-3’ |
| ANP | 5’-AATGCATCCTGCACCACC-3’ | 5’-CAGAGTGGGAGAGGCAAGAC-3’ |
| β-MHC | 5’-ATGTGCCGGACCTTGGAA-3’ | 5’-CCTCGGGTTAGCTGAGAGATCA-3’ |
| COL1A1 | 5’-GAGCGGAGAGTACTGGATCG-3’ | 5’-GTTCGGGCTGATGTACCAGT-3’ |
| TGFβ1 | 5’-GTTCGGGCTGATGTACCAGT-3’ | 5’-TCCTCCTTCTCTTCCCCTTC-3’ |
| CTGF | 5’-TGACCCCTGCGACCCACA-3’ | 5’-TACACCGACCCACCGAAGACACAG-3’ |
| Lysyl oxidase | 5’-TGCCAACACACAGAGGAGAG -3’ | 5’-CCAGGTAGCTGGGGTTTACA-3’ |
| MMP-2 | 5’-CTGATAACCTGGATGCCGTCGT-3’ | 5’-CCAGCCAGTCTGATTTGA-3’ |
| MMP-9 | 5’-TGAATCAGCTGGCTTTTGTG-3’ | 5’-GTGGATAGCTCGGTGGTGTT-3’ |
| TIMP-1 | 5’-ACTCTTCACTGCGGTTCTGGGAC-3’ | 5’-GTCATAAGGGCTAAATTCATGGG-3’ |
| TIMP-2 | 5’-GGATTCCGGGAATGACATCTAT-3’ | 5’-TGCCTTTCCTGCAATTAGATA-3’ |
| F4/80 | 5’-TTTCCTCGCCTGCTTCTTC-3’ | 5’-CCCCGTCTCTGTATTCAACC-3’ |
| MCP-1 | 5’-AGGTCCCTGTCATGCTTCTG -3’ | 5’-TCTGGACCCATTCCTTCTTG -3’ |
| IL-6 | 5’-AGTTGCCTTCTTGGGACTGA-3’ | 5’-TCCACGATTTCCCAGAGAAC-3’ |

GAPDH: glyceraldehyde 3-phosphate dehydrogenase; ANP: atrial natriuretic factor; β-MHC: β-myosin heavy chain; COL1A1: collagen type 1, alpha 1; TGFβ1: transforming growth factor β 1; CTGF: connective tissue growth factor; MMP-2/9: matrix metalloproteinase 2/9; TIMP-1/2: tissue Inhibitors of Metalloproteinase-1/2; MCP-1: monocyte chemoattractant protein-1; IL-6: interleukin-6.
