# Supplementary material for: Opposite Effects of Gene Deficiency and Pharmacological Inhibition of Soluble Epoxide Hydrolase on Cardiac Fibrosis
Source: PLoS One. 2014 Apr 9;9(4):e94092. doi: 10.1371/journal.pone.0094092 (PMC3981766; doi:10.1371/journal.pone.0094092)
Supplement: Methods S1 — (DOC) [file pone.0094092.s005.doc]

**Methods S1**

**Analysis of Cardiac Function by Echocardiography**

Analysis of cardiac function involved M-mode and 2D measurements. The measurements represented the mean of 6 selected cardiac cycles from at least 2 separate scans performed in a random-blinded fashion, with papillary muscles used as a point of reference for consistency in the level of scan. End diastole was defined as the maximal left-ventricular diastolic dimension and end systole as the peak of posterior wall motion.

**Western Blot Analysis**

Heart extracts were resolved by 10% SDS-PAGE and transferred to a polyvinylidene fluoride (PVDF) membrane (Millipore). sEH proteins were detected by use of a polyclonal anti-sEH (Santa Cruz Biotechnology, Santa Cruz, CA), then a horseradish peroxidase-conjugated secondary antibody. Level of GAPDH protein was also measured as an internal control. The protein bands were visualized by the ECL detection system (Amersham, Arlington Heights, IL) and the densities of the bands were quantified and normalized against GAPDH by use of Scion Image software (Scion Corp., Frederick, MD).

**Quantitative Real-Time RT–PCR**

Total RNA was isolated from heart or cells by the Trizol reagent method (Invitrogen, Carlsbad, CA). Aliquots of 2 μg of total RNA were used for first-strand cDNA synthesis with M-MLV reverse transcriptase (Promega). The amplification reactions were in a volume of 20 μl consisting of synthesized cDNA, primers and EasyTaq PCR Mix (Transgen Biotech, Beijing). Eva Green was used to monitor amplification of DNA by the MX3000P qPCR detection system (Stratagene, Santa Clara, CA, USA). Fold change in mRNA concentration was calculated by the comparative C_T_ method. Gene expression was normalized to GAPDH levels. The sequences of primers are in Table S2. All primers were synthesized by Sangon Biotechnology Co. (Beijing).
